# Type III Secretion Effector VopQ of Vibrio parahaemolyticus Modulates Central Carbon Metabolism in Epithelial Cells

**DOI:** 10.1128/mSphere.00960-19

**Published:** 2020-03-18

**Authors:** Anh Quoc Nguyen, Takaaki Shimohata, Sho Hatayama, Aya Tentaku, Junko Kido, Thi Mai Huong Bui, Takashi Uebanso, Kazuaki Mawatari, Akira Takahashi

**Affiliations:** a Department of Preventive Environment and Nutrition, Institute of Biomedical Sciences, Tokushima University Graduate School, Tokushima, Japan; b Department of Food Microbiology and Molecular Biology, National Institute of Nutrition, Hanoi, Vietnam; University of Kentucky

**Keywords:** metabolomics, host-pathogen interaction, *Vibrio parahaemolyticus*, T3SS effector

## Abstract

Vibrio parahaemolyticus is a Gram-negative halophilic pathogen that frequently causes acute gastroenteritis and occasional wound infection. V. parahaemolyticus contains several virulence factors, including type III secretion systems (T3SSs) and thermostable direct hemolysin (TDH). In particular, T3SS1 is a potent cytotoxic inducer, and T3SS2 is essential for causing acute gastroenteritis. Although much is known about manipulation of host signaling transductions by the V. parahaemolyticus effector, little is known about the host metabolomic changes modulated by V. parahaemolyticus. To address this knowledge gap, we performed a metabolomic analysis of the epithelial cells during V. parahaemolyticus infection using capillary electrophoresis-time of flight mass spectrometry (CE-TOF/MS). Our results revealed significant metabolomic perturbations upon V. parahaemolyticus infection. Moreover, we identified that T3SS1’s VopQ effector was responsible for inducing the significant metabolic changes in the infected cells. The VopQ effector dramatically altered the host cell’s glycolytic, tricarboxylic acid cycle (TCA), and amino acid metabolisms. VopQ effector disrupted host cell redox homeostasis by depleting cellular glutathione and subsequently increasing the level of reactive oxygen species (ROS) production.

**IMPORTANCE** The metabolic response of host cells upon infection is pathogen specific, and infection-induced host metabolic reprogramming may have beneficial effects on the proliferation of pathogens. V. parahaemolyticus contains a range of virulence factors to manipulate host signaling pathways and metabolic processes. In this study, we identified that the T3SS1 VopQ effector rewrites host metabolism in conjunction with the inflammation and cell death processes. Understanding how VopQ reprograms host cell metabolism during the infection could help us to identify novel therapeutic strategies to enhance the survival of host cells during V. parahaemolyticus infection.

## Abstract (precis)

The metabolic response of host cells upon infection is pathogen specific, and infection-induced host metabolic reprogramming may have beneficial effects on the proliferation of pathogens. V. parahaemolyticus contains a range of virulence factors to manipulate host signaling pathways and metabolic processes. In this study, we identified that the T3SS1 VopQ effector rewrites host metabolism in conjunction with the inflammation and cell death processes. Understanding how VopQ reprograms host cell metabolism during the infection could help us to identify novel therapeutic strategies to enhance the survival of host cells during V. parahaemolyticus infection.

## INTRODUCTION

Vibrio parahaemolyticus is an aquatic Gram-negative bacterium and the causative agent of the acute gastroenteritis associated with the ingestion of raw seafood and water. Occasionally, V. parahaemolyticus causes wound infection and septicemia in immunocompromised individuals (1, 2). The pandemic strains of V. parahaemolyticus are an important public health concern, and climate change is linked to the increased incidence of V. parahaemolyticus outbreaks worldwide (3, 4). Clinical isolates of V. parahaemolyticus contain numerous virulence factors, including pore-forming thermostable direct hemolysin (TDH) toxin and two type III secretion systems (T3SSs) that enable the delivery of bacterial effectors into the eukaryotic host (5, 6).

T3SS1 is located in an ancestral region corresponding to the bacterial first chromosome and is present in both nonpathogenic and pathogenic strains. The T3SS2-carried genes are located on the pathogenicity islands (V. parahaemolyticus PAI [Vp-PAI]) in the second chromosome and are associated with infectious diarrhea in humans (7–9). T3SS1 is cytotoxic in mammalian cells and yeast and causes mortality in murine peritoneal and pulmonary infection models, whereas T3SS2 is necessary for *in vivo* enterotoxicity in the infant rabbit and mouse infection models (10–13).

To date, four effectors of T3SS1 have been identified (14, 15). In particular, the VopQ effector (V. parahaemolyticus 1680 [VP1680], or VepA) effector has been extensively studied and has been found to be both necessary and sufficient to induce a nonapoptotic form of cell death *in vitro* (16). VopQ provokes inflammatory responses through the mitogen-activated protein kinase/extracellular signal-regulated kinase (MAPK/ERK) pathway, leading to interleukin-8 (IL-8) secretion in infected Caco-2 cells (17, 18). VopQ activates NLRC3 (NOD-like receptor CARD domain-containing 3) inflammasome complexes but interferes with the activation of NLRC4 (NOD-like receptor CARD domain-containing 4) inflammasomes in bone marrow-derived macrophages (19). Biochemical studies showed that the VopQ effector disrupts lysosomal proton gradients by interacting with the Vo domain of vacuolar H+-ATPase (v-ATPase) and forming outward rectifying pores in the lysosomal membrane, thus causing the release of small (<3-kDa) molecules from the lysosomal lumen (20, 21). Consequently, VopQ inhibits host autophagic flux by preventing lysosomal acidification and membrane fusion (22). Recently, transcriptome analysis revealed that T3SS1 activates prosurvival responses and suppresses cell death networks in infected human fibroblasts (23).

Metabolomics, as an emerging analytical platform, has been applied to decipher host physiological perturbation and identify altered metabolic pathways during bacterial infections (24, 25). In this study, we investigated the dynamics of the metabolic processes of the epithelial cells during V. parahaemolyticus infection and the role of the VopQ effector of T3SS1 in modulating host cell metabolism using capillary electrophoresis-time of flight mass spectrometry (CE-TOF/MS). Here, we identified that the cytotoxic VopQ effector of T3SS1 is a metabolic disruptor, profoundly altering host metabolisms.

## RESULTS

### V. parahaemolyticus infection-induced distinct metabolome alteration in the infected host cell.

Clinically isolated V. parahaemolyticus contains three principal identified virulence factors, including TDH toxin as well as type III secretion system 1 (T3SS1), and type III secretion system 2 (T3SS2), located in bacterial chromosomes 1 and 2, respectively (7). To study the metabolomic responses of the Caco-2 human adenocarcinoma epithelial cell line to the virulence factors of V. parahaemolyticus, we infected Caco-2 cells with several V. parahaemolyticus mutant strains carrying specific virulence factors. The ΔvscN1-ΔvscN2 mutant strain derived from the RIMD2210633 clinical isolate contains double deletions of genes *vscN1* and *vscN1*, which encode structural components of the T3SS1 and T3SS2 secretion apparatuses, respectively. Similarly, The POR2 and POR3 strains derived from the parental POR1 strain contain double genetic deletions of *tdhAS* and have deletions of *vcrD1* (T3SS1) and *vcrD2* (T3SS2), respectively, which encode the inner membrane structural ring for T3SS apparatus. The resulting mutant strains, the ΔvscN1-ΔvscN2 (ΔT3SS1, ΔT3SS2), POR2 (ΔTDH, ΔT3SS1), POR3 (ΔTDH, ΔT3SS2), and POR4 (ΔTDH, ΔT3SS1, ΔT3SS2) mutants, were used in our first metabolomic analysis to assess their impacts on the infected Caco-2 cell’s metabolomes. The bacterial strains used in this study and their phenotypic descriptions are summarized in Table 1 and Table 2.

**TABLE 1:** Strains and plasmids used in this study

Strain or plasmid	Genotype	Description^*a*^	Source or reference
Strains			
V. parahaemolyticus RIMD2210633	Wild type	Clinical isolate; KP positive, serotype O3: K6	7
V. parahaemolyticus vscN1-vscN2	Δ*vscN1* Δ*vscN2*	T3SS1- and T3SS2-deficient strain; *vscN1* and *vscN2* encoding a cytoplasmic protein of the T3SS apparatus deletion mutant derived from the wild-type strain	51
V. parahaemolyticus POR2	Δ*tdhAS* Δ*vscD1*	TDH toxin- and T3SS1-deficient strain: *vcrD1* needle apparatus gene deletion from POR-1 strain	51
V. parahaemolyticus POR3	Δ*tdhAS* Δ*vscD2*	TDH toxin- and T3SS2-deficient strain: *vcrD2* needle apparatus gene deletion derived from POR-1 strain	51
V. parahaemolyticus POR4	Δ*tdhAS* Δ*vcrD1* Δ*vcrD2*	TDH toxin-, T3SS1-, and T3SS2-deficient mutant derived from POR-1 in which the T3SS1 needle apparatus gene (*vcrD1*) and T3SS2 needle apparatus gene (*vcrD2*) were deleted	51
V. parahaemolyticus ET4	Δ*tdhAS* Δ*vscD2* Δ*VP1683* Δ*VP1686* Δ*VPA0450*	VP1680-containing strain; mutant strain deleted of other T3SS1 effectors; ΔVP1683, ΔVP1686, ΔVPA0450 derived from POR-3 strain	This study
V. parahaemolyticus S1-ENM	Δ*tdhAS* Δ*vscD2* Δ*VP1680* Δ*VP1683* Δ*VP1686* Δ*VPA0450*	T3SS1 effector-deficient strain; mutant strain deleted all T3SS1 effectors; ΔVP1680, ΔVP1683, ΔVP1686, ΔVPA0450 derived from POR-3 strain	This study
E. coli *SM10 λpir*	*thi thr leu tonA lacY supE recA*:: *RP4-2Tc*::*Mu* λ*pir R6K*	Plasmid mobilization strain	Laboratory collection
E. coli DH5α		TOPO subcloning	Laboratory collection

Plasmid			
pYAK		R6K-ori suicide vector containing *sacB* gene for counterselection, Cm^r^	51
pSA19CP^r^		Complement vector for V. parahaemolyticus	53
pSA-vp1680		Complement vector, expressing the open reading frame of gene *vp1680* controlled by a *tdhA* promoter, Cm^r^	This study

a Cm^r^, chloramphenicol resistant.

**TABLE 2:** Phenotypes of V. parahaemolyticus strains derived from the RIMD2210633 clinical isolate used in this study

Strain	TDH	T3SS1				T3SS2
		*VopQ*	*VopR*	*VopS*	*VPA0450*	
RIMD2210633	**+**	**+**	**+**	**+**	**+**	**+**
vscN1-vscN2	+	−	−	−	−	−
POR2	−	−	−	−	−	**+**
POR3	−	**+**	**+**	**+**	**+**	−
POR4	−	−	−	−	−	−
ET4	−	**+**	−	−	−	−
S1-ENM	−	−	−	−	−	−

**FIG 1:**
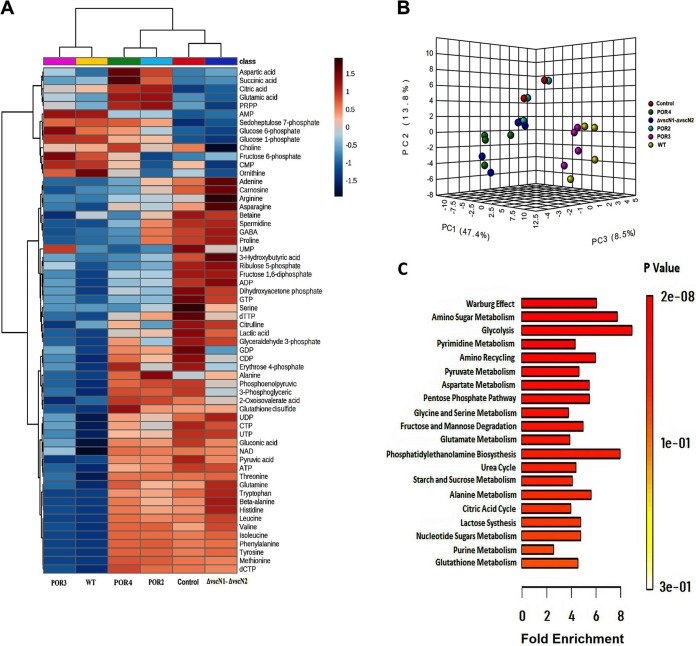
Multivariate analyses revealed a specific signature for Vibrio parahaemolyticus infection associated with T3SS1. Metabolites were extracted from Caco-2 cells infected with different V. parahaemolyticus strains at 120 min postinfection (MOI = 50:1; *n *= 4). (A) Heat map and hierarchical clustering of Caco-2 metabolomes according to the infection strains and metabolite abundances, showing the differences and similarities of the metabolic states of Caco-2 cells infected with V. parahaemolyticus strains. The metabolite abundances were scaled on a range of −1.5 to 1.5 and clustered according to Euclidean distance. (B) Three-dimensional (3D) principal-component-analysis (PCA) score plots showing distinct clusterization of Caco-2 cell metabolites infected with the control group (sham infection); the POR4 and ΔvscN1-ΔvscN2 mutant strains; the POR2 and POR3 mutant strains; and the wild-type (WT) strain. (C) Metabolite set enrichment analysis (MSEA) showed 20 significantly (*P < *0.05) enriched biological pathways associated with V. parahaemolyticus infection. Color intensity (white to red) indicates the increasing statistical significance of pathway enrichment analysis. The data on the graph corresponding to the *y* axis show the −log of *P* values from pathway enrichment analysis, and the data corresponding to the *x* axis show the −log of *P* values from pathway topology analysis. PRPP, phosphoribosyl diphosphate.

The infections were performed at a multiplicity of infection (MOI) of 50 bacterial cells per infecting Caco-2 cell to ensure full exposure of the infected cell population, and the total metabolome was extracted at 120 min postinfection to avoid excessive cytotoxicity causing leakages of cellular metabolites (see Fig. S1 in the supplemental material). CE-TOF/MS identified 87 metabolites matching with the validated standards, and the changes in the relative levels of quantified metabolites seen upon infection were visualized by heat map hierarchical clustering as shown in Fig. 1A. Principal-component analysis (PCA) (Fig. 1B) was performed to demonstrate further the distinct clusters of metabolites present in the different infection groups. The combination of the three components shown cumulatively explains 69.7% of the variation in the metabolite data set.

10.1128/mSphere.00960-19.1 FIG S1 (A) Metabolomics analysis workflow of Caco-2 cells infected with Vibrio parahaemolyticus strains. (B) Cytotoxic activity (percent LDH release) of Caco-2 cells infected with different Vibrio parahaemolyticus strains for the WT strain or the isogenic mutants at a multiplicity of infection (MOI) of 50:1 at 3 h postinfection. (C) Cell viability (Trypan blue exclusion staining) of Caco-2 cells infected with different Vibrio parahaemolyticus strains for the WT strain or the isogenic mutants at a multiplicity of infection (MOI) of 50:1 at 3 h postinfection. The strains tested were the WT (RIMD2210633), POR2 (Δ*tdhAS* Δ*vscD1*), POR3 (Δ*tdhAS* Δ*vscD2*), vscN1-vscN2 (Δ*vscN1*-Δ*vscN2*), and POR4 (Δ*tdhAS* Δ*vscD1*, Δ*vscD2*). The error bars indicate standard deviations of results from triplicate samples (*n* = 3). Download FIG S1, TIF file, 1.2 MB. Copyright © 2020 Nguyen et al. 2020 Nguyen et al. This content is distributed under the terms of the Creative Commons Attribution 4.0 International license.

To determine which host biochemical pathways were significantly altered by V. parahaemolyticus infection, we performed functional metabolite set enrichment analysis (MSEA) to identify overrepresented metabolites in the cells infected with the V. parahaemolyticus wild-type strains compared to the control group (sham infection) with human metabolites’ library databases using MetaboAnalyst Suite 4.0 (26). The MSEA data are presented according to the scores from enrichment analysis (*y* axis) and topology analysis (*x* axis) (Fig. 1C). The infection of Caco-2 cells with V. parahaemolyticus resulted in drastic changes in the multiple metabolites involved in glycolysis and its associated pathways, amino acid and nucleotide metabolism, and the tricarboxylic acid cycle (TCA) cycle metabolites (Fig. 1C). Overall, the metabolomic response to V. parahaemolyticus infection showed a strong correlation with the presence of the T3SS1 virulence factor (POR3 and wild type [WT]), and the metabolic profiles of cells infected with the POR4 strain, POR2 strain, or ΔvscN1–ΔvscN2 strain were more highly correlated with those seen with the control group. Consequently, although there was divergence in the metabolomes of cells infected with the POR4, ΔvscN1–ΔvscN2, and POR2 strains in the comparisons with the control group (sham infection) (Fig. 1B), in the context of metabolic analysis, it appeared that T3SS1 contributed the most significant metabolic perturbation observed in the infected Caco-2 cells.

### VopQ, a T3SS1 effector, rewrites host cell metabolomes.

T3SS1 translocates four identified protein effectors, including VopQ (VP1680), VopR (VP1683), VopS (VP1686), and VPA0450, working in tandem to induce autophagosome accumulation, plasma membrane blebbing, cell rounding, and, eventually, cell death (17, 18). Since VopQ is known to induce cell cytotoxicity and lysosomal dysfunctions, we hypothesized that VopQ is a metabolic modifier of T3SS1 that underlies the host metabolic perturbation. Next, we constructed a V. parahaemolyticus ET4 strain, derived from the parental POR3 strain, which contained only a single VopQ effector (ΔVP1683, ΔVP1686, and ΔVPA0450) and S1-ENM, a V. parahaemolyticus T3SS1 effector-deficient strain (ΔVP1680, ΔVP1683, ΔVP1686, and ΔVPA0450).

**FIG 2:**
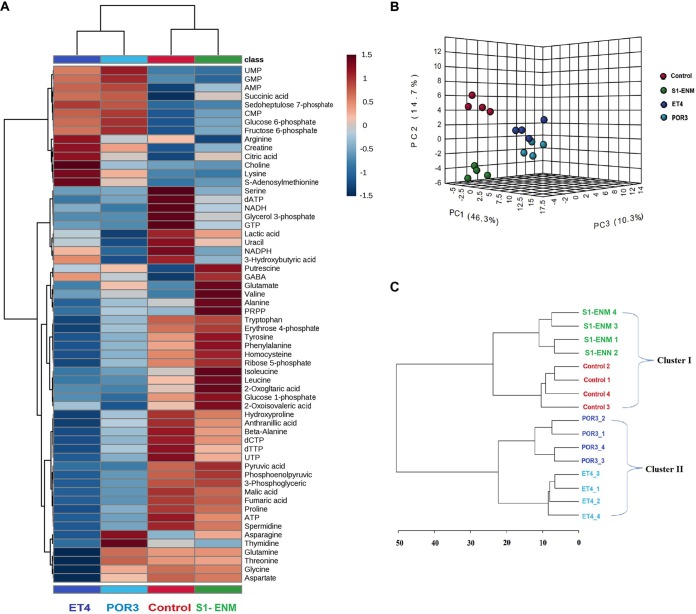
Multivariate analyses showed a specific metabolic signature associated with VopQ. Metabolite was extracted from Caco-2 cells infected with different S1-ENM and ET4 strains at 90 min postinfection (MOI = 50:1; *n *= 4). (A) Heat map and hierarchical clustering of Caco-2 metabolomes according to the infection strains and metabolite abundances showing the similarities of the metabolic states of Caco-2 cells infected with POR3 and ET4 strains compared to strain S1-ENM and the control. The metabolite abundances were scaled on a range of −1.5 to 1.5 and clustered according to Euclidean distance. (B) Three-dimensional (3D) principal-component analysis (PCA) score plots showed distinct clusterization of Caco-2 cell metabolites infected with POR3 and ET4 compared with the control group (sham infection) and S1-ENM. (C) Hierarchical clustering analysis was performed according to Euclidean distance between the control group (sham infection); POR3, ET4, and S1-ENM showed two district clusters.

We performed a metabolomic analysis of Caco-2 cells infected with VopQ-positive (VopQ^+^) strains (POR3 and ET4) and with S1-ENM, a VopQ-deficient strain. We observed the profound metabolomic alterations at 90 min postinfection in the cells infected with the T3SS1^+^ strain (POR3 and ET4) (Fig. 2A). In particular, principal-component analysis (PCA) showed clear discrimination between the Caco-2 cells infected with the ET4-infected and POR3-infected cells, compared to the control (sham infection) and S1-ENM strain-infected cells. The combination of the three components shown cumulatively explained 71.3% of the variation in the metabolite data set (Fig. 2B). Also, hierarchical cluster analysis of the infection group’s metabolite data set by Euclidean distance showed two distinct clusters, namely, cluster I (S1-ENM and control) and cluster II (POR3 and ET4) (Fig. 2C). Thus, our results suggest that VopQ of T3SS1 was responsible for the host metabolic change in the Caco-2 infection model.

Additionally, to monitor the onset of metabolomic changes during the infection, we performed a time-series analysis of Caco-2 cells infected with ET4 and S1-ENM strains. We observed progressively increasing metabolomic alterations starting from 90 min postinfection but not from the initial 45 min postinfection in ET4 strain-infected cells compared to the control results and S1-ENM infection (Fig. S2 in the supplemental material).To further confirm these results, we analyzed the metabolomes of cells of human cervix carcinoma cell line INT-407 (a HeLa derivative) infected with ET4 and S1-ENM strains. Consistent with the previous metabolomic analysis results from Caco-2 cells, ET4 induced a significant metabolomic change in INT-407 cells, and VopQ effector-deficient strain S1-ENM showed a metabolic pattern similar to that of the control (Fig. S3 in the supplemental material).

10.1128/mSphere.00960-19.2 FIG S2 Multivariate analyses showed a specific metabolic signature associated with VopQ. Metabolite was extracted from Caco-2 cells infected with different S1-ENM and ET4 strains at 45 min, 90 min, and 150 min postinfection (MOI = 50; *n* = 3). A heat map and hierarchical clustering of Caco-2 metabolomes are represented according to comparisons of metabolite abundances between the control (sham infection) and infections by strains S1-ENM and ET4, showing the differences and similarities of the metabolic patterns of the Caco-2 cells at different time points. The metabolite abundances were scaled in a range of −3 to 3 and clustered according to the Euclidian distance matrix. Download FIG S2, TIF file, 1.8 MB. Copyright © 2020 Nguyen et al. 2020 Nguyen et al. This content is distributed under the terms of the Creative Commons Attribution 4.0 International license.

10.1128/mSphere.00960-19.3 FIG S3 Multivariate analyses showed that the VP1680 effector repressed the central metabolism of INT-407 cells. Metabolite was extracted from INT-407 cells infected with different V. parahaemolyticus strains at 90 min postinfection (MOI = 10:1; *n* = 4). The heat map represents results of hierarchical clustering analysis, with statistically significant differences in relative metabolite abundances between the control (sham infection) and infections by strains S1-ENM and ET4. The metabolite abundances were scaled in a range of −3 to 3 and clustered according to the Euclidean distance matrix. Download FIG S3, TIF file, 1.6 MB. Copyright © 2020 Nguyen et al. 2020 Nguyen et al. This content is distributed under the terms of the Creative Commons Attribution 4.0 International license.

### The VopQ effector repressed host cell energy metabolism.

**FIG 3:**
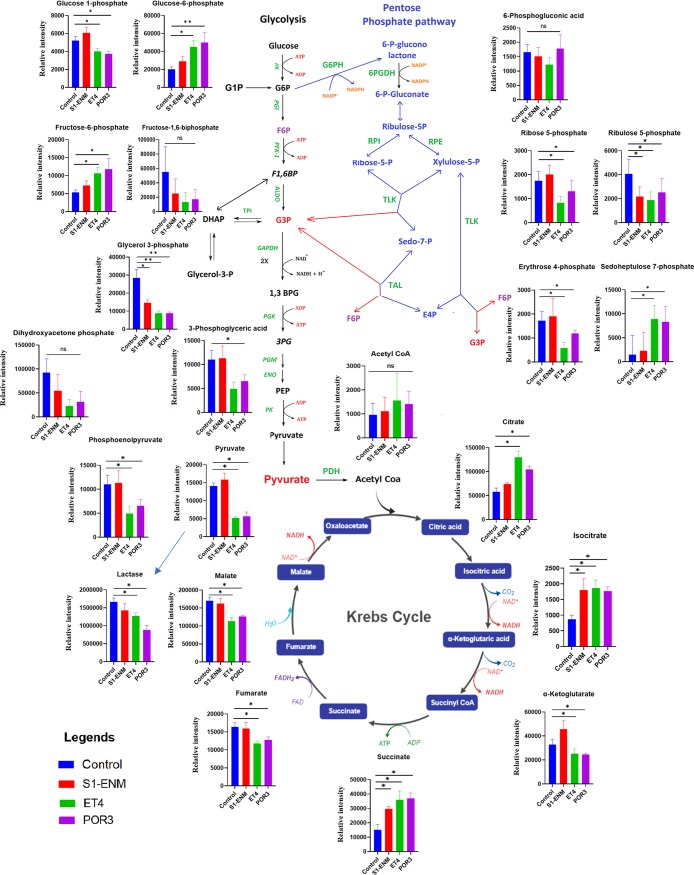
VopQ rerouted Caco-2 cell metabolites from glycolysis to PPP metabolites. The relative abundances of glycolytic and PPP metabolites of Caco-2 cells infected with different V. parahaemolyticus mutants extracted 90 min postinfection were determined. Means ± standard deviations (SD) are shown for results from 4 biological replicates. One-way ANOVA with Dunnett’s multiple-comparison test was applied to the S1-ENM, ET4, and POR3 groups for comparison to the control group (*, *P < *0.05; **, *P < *0.01; ns, not significant). The *y* axis data represent the relative abundances in peak intensity (concentration) of the identified metabolites. The black letters, light blue letters, and white letters on a dark blue background represent the metabolic intermediates from glycolysis, the pentose phosphate pathway, and the TCA cycle, respectively. The green and orange letters signify enzymes and cofactors that catalyze the corresponding metabolic pathways.

Among the metabolites that were determined by the CE-TOF analytical system, we observed 2-to-3-fold-increased levels of upper glycolytic intermediates such as glucose 6-phosphate (G6P) and fructose 6-phosphate (F6P) in Caco-2 cells infected by V. parahaemolyticus POR3 and ET4 strains but not in those infected by the S1-ENM strain at 90 min postinfection (*P < *0.05) (Fig. 3). Interestingly, the levels of subsequent downstream glycolytic intermediates were altered in Caco-2 cells infected with ET4 or POR3 compared to S1-ENM and the control. In those infected with strains ET4 and POR3, we found significant reductions of downstream glycolytic intermediates, including fructose 1,6-phosphate (F-1,6-P), 3-phosphoglycerate (3GP), phospho(enol)pyruvic acid (PEP), and pyruvic acid (PYR) (*P < *0.05) (Fig. 3), compared to Caco-2 cells infected with the S1-ENM mutant or with the control. However, we were unable to detect glyceraldehyde 3-phosphate and 2-phosphoglycerate, perhaps because of their high rate of turnover or low stability. Also, the level of the end product of glycolysis, lactate, was lower in ET4-infected and POR3-infected cells than in those infected with S1-ENM or the control (*P < *0.05). These data suggested that the activity of the VopQ effector of V. parahaemolyticus halted glycolytic cycles in the infected cells.

The accumulation of the upper and the depletion of the lower glycolytic intermediates in Caco-2 cells infected with ET4 and POR3 strains indicated the presence of a bottleneck in glycolytic flux at the step catalyzed by enzyme phosphofructokinase-1 (PFK-1) that results in conversion of G6P to F-1,6-P. The accumulation of F6P and decreased downstream glycolytic metabolites in ET4-infected and POR3-infected cells were correlated with the increased levels of pentose phosphate pathway (PPP) intermediates such as sedoheptulose-7-phosphate, which indicated a possible metabolic rerouting from glycolysis to PPP. Since phosphofructokinase-1 (PFK-1), a tetramer that is encoded by PFK-M (muscle), PFK-L (liver), and PFK-P (platelets), functions as the gatekeeper to glycolysis by catalyzing the phosphorylation of F6P to F1,6-P, we determined whether VopQ directly inhibits cellular PFK-1 activity in infected Caco-2 cells by enzymatic assay (27). Unexpectedly, the PFK-1 activities of Caco-2 cells infected with POR3 and ET4 were equivalent to those of cells infected with S1-ENM, and the control results indicated that VopQ indirectly inhibited PFK-1 function (Fig. S4 in the supplemental material).

10.1128/mSphere.00960-19.4 FIG S4 Relative phosphofructokinase activities of Caco-2 cells infected with the POR3, ET4, and S1-ENM strains and the control (sham infection) extracted at 2 h postinfection. No statistically significant difference was detected by one-way ANOVA with Dunnett’s multiple-comparison test applied to the S1-ENM, ET4, and POR3 groups for comparison to the control group (*n* = 4). Download FIG S4, TIF file, 0.3 MB. Copyright © 2020 Nguyen et al. 2020 Nguyen et al. This content is distributed under the terms of the Creative Commons Attribution 4.0 International license.

**FIG 4:**
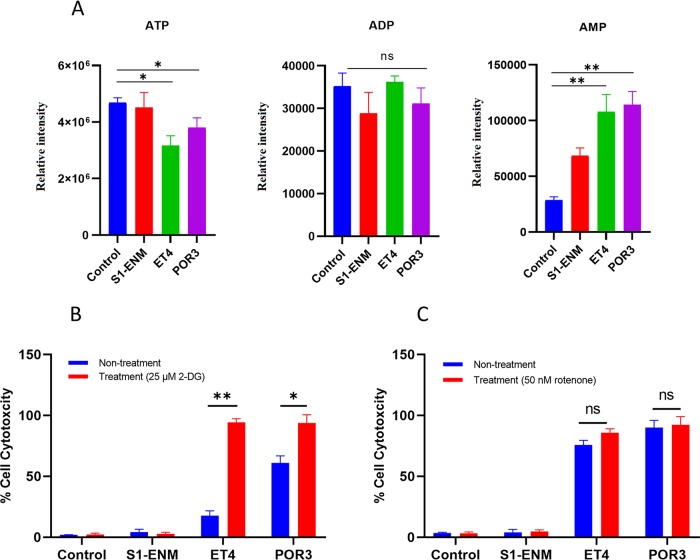
VopQ disrupted cellular biogenetics. (A) The relative abundances of ATP, ADP, and AMP metabolites of Caco-2 cells infected with different V. parahaemolyticus mutants measured by CE-MS at 90 min postinfection. Means ± SD are shown for results from four biological replicates. One-way ANOVA with Dunnett’s multiple-comparison test was applied to the S1-ENM, ET4, and POR3 groups for comparisons to the control group (*, *P < *0.05; **, *P < *0.01). Caco-2 cell cytotoxicity at 4 h with different V. parahaemolyticus mutants was measured by LDH release assay. (B and C) Caco-2 cells were treated with (B) 25 mM 2-deoxyglucose (2-DG) and (C) 50 nM rotenone. Statistical analysis was performed using Student's *t* test (*, *P < *0.05; **, *P < *0.01), with means ± SD shown for results from three biological replicates.

We observed dramatic alterations of the TCA cycle’s intermediary metabolism in the infected cells with VopQ^+^ strains (POR3 and ET4). Pyruvate, which is the end product of glycolysis and is converted to acetyl coenzyme A (acetyl-CoA), was severely depleted in the infected cells with POR3 and ET4. Pyruvate contributes energy to the living cells by its conversion to acetyl-CoA, a primary fuel of the TCA cycle. Even though we observed a steeply decreased level of pyruvate, the level of acetyl-CoA was slightly reduced through the reduction was not statistically significant in the POR3-infected and ET4-infected cells (Fig. 3). Moreover, the levels of citrate, isocitrate, and succinate increased in cells infected with VopQ^+^ strains whereas the levels of malate, fumarate, and α-ketoglutarate decreased (Fig. 3). Citrate is a potent inhibitor of phosphofructokinase-1, and the intracellular accumulation of citrate can lead to the inhibition of glycolysis and the increase of F6P and G6P levels, as seen in our results (Fig. 3) (27, 28). Expectedly, the cellular nucleoside triphosphates (ATP) were decreased in level in conjunction with a significantly increased level of nucleoside monophosphates (AMP) (Fig. 4A). To determine whether the deprivation of pyruvate was responsible for the depletion of cellular ATP, we treated Caco-2 cells with ethyl pyruvate (EP), a cell-permeable derivative of pyruvic acid that can be readily converted to acetyl-CoA (29). Ethyl pyruvate is an effective reactive oxygen species (ROS) scavenger with anti-inflammatory properties and is cytoprotective (30). Administration of 5 mM (or 10 mM) ethyl pyruvate did not restore ATP level and was not cytoprotective for VopQ^+^ strain-infected Caco-2 cells (Fig. S7 in the supplemental material). This limited result suggested that the deprivation of pyruvate *per se* is unlikely to contribute to the reduction of cellular ATP through the TCA cycle.

Glycolysis and oxidative phosphorylation are central ATP-generating biochemical pathways in the cells. To examine the contribution of energy-generating pathways to the survival of Caco-2 cells during infection, we pretreated Caco-2 cells with the glycolytic inhibitor 2-deoxy-d-glucose (2-DG) and with rotenone, a mitochondrial respiratory chain complex I inhibitor. The result showed that inhibition of glycolysis but not mitochondrial oxidative phosphorylation sensitized Caco-2 cells to VopQ-mediated cell death (Fig. 4B and C). Furthermore, Caco-2 cells cultured in glucose-free Dulbecco’s modified Eagle’s medium (DMEM) 1 day after reaching confluence or Caco-2 cells cultured in (glucose-free) galactose-DMEM, which is known to enhance oxidative phosphorylation but to reduce glycolytic flux, showed increased susceptibility to VopQ (31, 32) (Fig. S5 in the supplemental material). In summary, our data suggested that VopQ repressed cellular energy metabolism in Caco-2 cells preceding the cellular destruction.

10.1128/mSphere.00960-19.5 FIG S5 Caco-2 cells were cultured in the high-glucose medium DMEM (4.5 mg/ml glucose) for 4 days. For glucose-free medium culture, after reaching confluence, the cells in the medium containing glucose were then maintained in glucose-free DMEM for 1 day before the experiment. For the galactose medium culture, Caco-2 cells were cultured in galactose medium for 4 passages in 20 days. The fifth cell passage was selected for the experiment. The conditions used to determine the cytotoxicity of Caco-2 cells infected with different V. parahaemolyticus strains were as follows: 4.5 mg/ml DMEM, glucose-free medium, 4.5 mg/ml galactose medium for 4 h. Means ± standard deviations (SD) are shown for results from 4 biological replicates. Student’s *t* test was performed for paired comparisons for treatments in each group (**, *P* < 0.01). Download FIG S5, PDF file, 0.1 MB. Copyright © 2020 Nguyen et al. 2020 Nguyen et al. This content is distributed under the terms of the Creative Commons Attribution 4.0 International license.

### VopQ alters host intracellular amino acid and induced reactive oxygen species (ROS) production.

**FIG 5:**
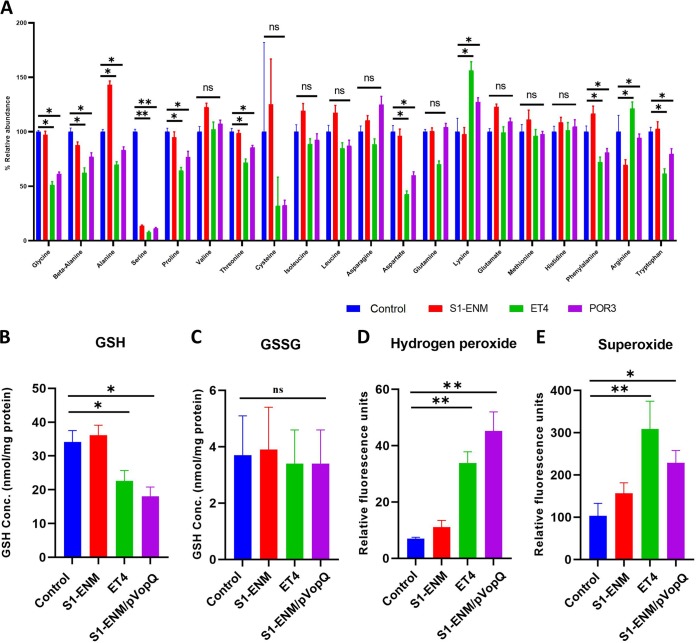
VopQ disrupted cellular amino acid hemostasis. (A) Percent relative abundances of amino acids of Caco-2 cells infected with different V. parahaemolyticus mutants measured by CE-MS at 90 min postinfection. Means ± SD are shown for results from four biological replicates. One-way ANOVA with Dunnett’s multiple-comparison test was applied to the S1-ENM, ET4, and POR3 groups for comparisons to the control group (*, *P < *0.05; **, *P < *0.01). (B and C) Cellular glutathione and glutathione disulfide (GSSG) concentrations of Caco-2 cells infected with different V. parahaemolyticus mutants for 2 h. (D and E) Relative levels of hydrogen peroxide (H_2_O_2_) and superoxide (O_2_^−^) of Caco-2 cells infected with different V. parahaemolyticus mutants for 2 h. Statistical analysis by one-way ANOVA with Dunnett’s multiple-comparison test was applied to the S1-ENM, ET4, and POR3 groups for comparisons to the control group for 4 biological replications (*, *P < *0.05; **, *P < *0.01).

Throughout V. parahaemolyticus infection, we observed significant changes in amino acid abundances in the infected cells which were strongly associated with the presence of the VopQ effector. At 90 min postinfection, we also detected that VopQ induced increases in intracellular abundances of lysine and arginine but decreased the intracellular abundances of glucogenic amino acids such as glycine, beta-alanine, serine, proline, aspartate, and the two essential amino acids tryptophan and threonine (Fig. 5A). The level of alanine, a principal metabolite of pyruvate metabolism principally produced via pyruvate transamination, was also markedly reduced in cells infected with VopQ^+^ strains (*P < *0.05) (Fig. 5A). In addition, our metabolomic analysis indicated that the level of the cellular antioxidant glutathione (GSH) but not that of glutathione disulfide was reduced in cells infected with the VopQ^+^ effector strains compared to S1-ENM infections that lacked VopQ. The complementation of the VopQ gene on a plasmid restored the reduced GSH-depleting phenotype of S1-ENM (Fig. 5B and C). This result indicated that the presence of the VopQ effector was necessary and sufficient to deplete the epithelial cell’s GSH pool. Indeed, VopQ-induced reduction of GSH was accompanied by a steep rise in the levels of cellular hydrogen peroxide (H_2_O_2_) and superoxide (O_2_^−^), measured by fluorescence spectroscopic assay (Fig. 5D and E).

Since glycine and cysteine availability is the rate-determining factor in GSH biosynthesis, we tested the hypothesis that the reduction of cytosol glycine and cysteine jointly determined the glutathione synthesis rates in infected cells. Supplementation of glycine or cell-permeated *N*-acetylcysteine or both in the culture medium did not lead to the restoration of the total GSH content in strain ET4-infected cells (Fig. S6 in the supplemental material). On the other hand, there was no GSH detected in the culture medium during the infection, indicating that GSH efflux was unlikely to be responsible for the depletion of GSH content. Taken together, our results suggested that the VopQ-induced GSH depletion and the subsequently increased level of reactive oxygen species (ROS) in infected cells occurred independently of the variations in the glycine and cysteine levels.

10.1128/mSphere.00960-19.6 FIG S6 Effects of glycine and N-acetyl cysteine (NAC) on the GSH level. Caco-2 cells were incubated with 0.5 mmol/liter glycine and/or 0.5 mmol/liter NAC for 8 h prior to starting an infection. GSH content was measured by colorimetric assay at 3 h postinfection. Results are expressed as means, and SD were calculated from results from 4 biological replications. No statistically significant differences were found by one-way ANOVA with Dunnett’s multiple-comparison test between treatments with ET4 infection. One-way ANOVA with Dunnett's multiple-comparison test showed statistically significant differences in GSH contents between the mocked infection and all infected groups (*, *P* < 0.05). Download FIG S6, TIF file, 0.4 MB. Copyright © 2020 Nguyen et al. 2020 Nguyen et al. This content is distributed under the terms of the Creative Commons Attribution 4.0 International license.

10.1128/mSphere.00960-19.7 FIG S7 Effects of ethyl pyruvate on ATP level and cell cytotoxicity in Caco-2 cells. Caco-2 cells were incubated with 5 mM and 10 mM of ethyl pyruvate for 2 h before and continuously during the infection (MOI = 50:1) with POR3. (A) Cellular ATP content was measured by a luciferase assay at 2 h postinfection. (B) Cytotoxic activity (percent LDH release) of Caco-2 cells at 6 h postinfection. Results are expressed as means, and SD were calculated from results from 4 biological replications. No statistically significant differences were found by one-way ANOVA performed with Dunnett’s multiple-comparison test in comparisons between treatments with POR3 infection. Download FIG S7, TIF file, 0.1 MB. Copyright © 2020 Nguyen et al. 2020 Nguyen et al. This content is distributed under the terms of the Creative Commons Attribution 4.0 International license.

## DISCUSSION

In this study, we explored the metabolomic landscape of epithelial cells infected with V. parahaemolyticus. We determined that a bacterial T3SS1 effector, VopQ, induced a widespread metabolic perturbation in the infected cells, profoundly altering host central carbon metabolism and amino acid metabolism. During V. parahaemolyticus infection, VopQ inhibits the activation of the host NLRC4 inflammasome but triggers the assembly of NLRC3 inflammasomes by activating caspase-1 processing of pro-IL-1b and pro-IL-18 and, eventually, the secretion of proinflammatory cytokines (19). Cellular metabolism plays a central role in regulating immune responses and inflammation in immune cells (33, 34). Several studies have suggested that inflammasome activation is modulated by several glycolytic enzymes (35). For example, inhibiting hexokinase-1 or pyruvate kinase (PKM2) suppresses NLRP3 inflammasome activation in macrophages (36, 37). While other studies reported inhibition of the glycolytic enzymes by 2-deoxyglucose (2-DG) treatment, glyceraldehyde 3-phosphate dehydrogenase (GAPDH) and α-enolase can trigger NLRP3 inflammasome activation (38, 39). These contradictory results illustrate the mechanistic complexity and plasticity of cellular metabolism. In *Salmonella*-infected macrophages, the level of cellular glycolysis is diminished because of the uptake of host cell glucose by *Salmonella* in conjunction with SPI-1 T3SS-dependent NLRP3 activation (40). Mechanistically, a Salmonella enterica serovar Typhimurium mutant strain lacking the TCA enzyme aconitase induced rapid NLRP3 inflammasome activation by inducing bacterium-derived citrate accumulation, leading to the upstream accumulation of F6P and G6P (40). Our results indicated that VopQ-mediated inhibition of host cell glycolysis may be linked to the activation of NLRC3 inflammasomes in macrophages (19). In addition, our results showed that the decrease in glycolysis flux increased the sensitivity to VopQ-mediated cell death. These findings suggest that the VopQ effector interferes with cellular energy metabolism and the survival of the host cells.

Cells infected with VopQ showed decreased metabolism of amino acids, including several nonessential amino acids and some metabolites involved in the anabolic pathways of these amino acids. Amino acids play a pivotal role in regulating physiology and cell proliferation. Consequently, variations in cellular amino acid metabolism could substantially influence the outcome of an infection. Indeed, bacterial, parasitical, and viral infections have been associated with significant metabolic alterations in mammalian hosts (41, 42). For instance, enterotoxigenic Escherichia coli (ETEC) infection decreases isoleucine levels in piglet serum and reduces the concentrations of nonessential amino acids, including glutamine, asparagine, citrulline, and ornithine, but increases the levels of glycine and GABA (gamma aminobutyric acid) (43). During invasive *Salmonella* or *Shigella* infections, levels of intracellular amino acids of epithelial cells were acutely depleted, leading to the inhibition of mTOR activity and the activation of autophagy (44). Indeed, VopQ is capable of rapidly inducing autophagy in both phagocytic and nonphagocytic cells (19, 45).

The alteration of the intracellular amino acids induced by VopQ can be a result of changes in amino acid metabolism or amino acid transport or both. Both glycolysis and the TCA cycle provide indispensable carbon skeletons, including 3-phosphoglyceric acid, pyruvic acid, and α-ketoglutarate, for the biosynthesis of nonessential amino acids. Through a cascade of enzymatic reactions, chiefly those involving transaminases and aminotransferases, these carbon donors are converted into the corresponding amino acids serine, glycine, and cysteine (3-phosphoglyceric acid); alanine (pyruvic acid); and aspartic acid and glutamic acid (α-ketoglutarate) (46). Therefore, the *de novo* biosynthesis of amino acids strictly requires the availability of a carbon donor or a structurally related amino acid. The decreased level of nonessential amino acids in infections with VopQ^+^ strains is consistent with the severe depletion of intermediates of the glycolytic and the TCA cycles, namely, 3-phosphoglyceric acid, pyruvic acid, and α-ketoglutarate. Although we cannot exclude the possibility that VopQ can alter the rate of amino acid transport in the infected cells, it is likely that the reduction of nonessential amino acids induced by VopQ was due to a lack of carbon donor precursors.

Another important finding in our study was that VopQ depleted the host’s cellular glutathione levels and induced oxidative stress. Glutathione is a tripeptide, consisting of glycine, cysteine, and glutamate, that participates in many critical cellular functions in mammalian cells (47, 48). Glutathione is predominantly found in the cytosol, mainly in the reduced state (GSH) and less often in its oxidized state, glutathione disulfide (GSSG). Together with other antioxidant enzymes, the GSH/GSSG redox system safeguards the cellular oxidative balance (49). GSH plays a major role in the removal of many reactive species such as superoxide (O_2_^−^) and hydrogen peroxide (H_2_O_2_) through peroxidase oxidization of GSH to GSSG and H_2_O. The depletion of GSH is a marker the associated pathological state of a disease and the development of destructive cellular processes such as apoptosis and necrosis (50).

Our study had several limitations. First, we measured the abundance of selected metabolites and therefore captured only a metabolomic snapshot at a given time of sampling but not the metabolic turnover rate, which represents the dynamic status of cellular biochemical processes. Consequently, we were unable to elucidate the exact nature of the metabolic flux of the responses of host cells to V. parahaemolyticus infection. Second, our study used Caco-2 and INT-407 cell lines, which are established cancer cell lines, as the infected host cells. Those cell lines are known to be metabolically altered to favor glycolytic metabolism over oxidative phosphorylation, even under aerobic conditions. Thus, this inherent metabolic abnormality can mask pathogen-induced host interaction. Additional studies with other primary cells are warranted to confirm further the general relevance of these metabolomic patterns observed in Caco-2 cells. In summary, we identified that the V. parahaemolyticus VopQ effector subverted Caco-2 cells’ energy metabolism, amino acid balance, and redox hemostasis. Boosting the cellular bioenergetic state can be a promising target to enhance cellular survival during V. parahaemolyticus infection.

## MATERIALS AND METHODS

### Bacterial strains and culture conditions.

V. parahaemolyticus strain RIMD2210633 (Kanagawa phenomenon [KP] positive, serotype O3: K6) was used as the standard strain. The bacteria were cultured in Luria–Bertani (LB) medium supplemented with 3% NaCl at 37°C. For the construction of deletion mutants, Escherichia coli DH5α and SM10λpir strains were used to mobilize plasmids into V. parahaemolyticus. Escherichia coli strains were cultured in LB medium supplemented with the following antibiotics at the indicated concentrations with shaking at 37°C: kanamycin 50 μg ml^−1^ and chloramphenicol 20 μg ml^−1^. The strains and constructed plasmids used in this study are listed in Table S1 in the supplemental material.

10.1128/mSphere.00960-19.8 TABLE S1 Primers list used in this study. Download Table S1, PDF file, 0.1 MB. Copyright © 2020 Nguyen et al. 2020 Nguyen et al. This content is distributed under the terms of the Creative Commons Attribution 4.0 International license.

### Construction of deletion mutants.

Deletions of mutant strains were constructed by a two-step allelic exchange method using POR3 as the parental strain, as previously described (51). Briefly, approximately 400-to-500-nucleotide fragments of DNA regions upstream and downstream of the targeted gene were amplified by PCR using the primers listed in Table S1 in the supplemental material. Primers 2 and 3 contained a 15-to-18-bp overlapped site to connect the 3′ end of the upstream fragment with the 5′ end of the downstream fragment. Two amplified DNA fragments were conjoined by the second round of overlapping PCR using primers 1 and 4. The amplified fragment was cloned by the use of blunt end TOPO cloning (Thermo Fisher Scientific) and propagated in E. coli DH5α. The inserted fragment containing a deletion cassette was excised by restriction digestion with BamHI and PstI and subcloned into the suicide vector pYAK1. The constructed plasmid was introduced into E. coli strain SM10λpir and was mobilized into the V. parahaemolyticus POR3 strain by conjugation. The crossing-over colonies were selected by counterselection on 10% thiosulfate-citrate-bile salts-sucrose (TCBS). Isolates with a deletion allele were confirmed by PCR and sequencing.

### Complementation of deleted genes.

Complementation of deleted genes was carried out as previously described (51). Briefly, the VP1680 coding region of VopQ was amplified by PCR and was subsequently cloned into pSA19CP-MCS, and the plasmid construct was introduced into the deletion mutant strains by electroporation.

### Cell culture.

Caco-2 cells and INT-407 cells were cultured in DMEM (Sigma-Aldrich) supplemented with 10% fetal bovine serum (Gibco BRL) and 50 μg/ml gentamicin (Sigma-Aldrich). Caco-2 cells and INT-407 cells were seeded at 4 × 10^5^ and 8 × 10^5^ cells/well, respectively, in a 6-cm-diameter cell culture plate. The cells were maintained at 37°C in a humidified atmosphere of 5% CO_2_, and the Caco-2 and INT-407 cells were cultured for 4 and 3 days, respectively, and used for subsequent experiments.

### Infection protocol.

The culture medium was replaced with fresh DMEM (without supplements) at 2 h before infection. For the first metabolomics analysis (results shown in Fig. 1), after overnight cultivation, bacteria were harvested by centrifugation at 6,000 rpm at room temperature and were resuspended in phosphate-buffered saline (PBS) and adjusted to an optical density at 600 nm (OD_600_) of 1.0. Bacteria were inoculated into the mammalian cells at a multiplicity of infection (MOI) of 50 bacterial cells to 1 Caco-2 cell (50:1), and the infections were allowed to proceed at 37°C in 5% CO_2_ at each time point.

For the second metabolomics analysis (results shown in Fig. 2), to ensure the uniform expression of VP T3SS1, overnight cultured bacteria were inoculated in DMEM in a humidified atmosphere containing 5% CO_2_ at 37°C for 6 h before the start of the experiment. Other subsequent steps were the same as described in the text above.

### Metabolite extraction and CE-TOF/MS analysis.

Cells were collected for metabolome analysis at the indicated infection times. Approximately 2.5 × 10^6^ cells were infected with each V. parahaemolyticus strain at each given time point at an MOI of 50:1. The supernatant was removed, and cells were washed twice with 5 ml of cold 5% mannitol solution. Metabolic activity was rapidly quenched by adding 0.5 ml methanol containing internal standards (100 μM methionine sulfone and camphor 10-sulfonic acid). Intracellular metabolites were extracted using a solvent extraction method by mixing homogenates with 400 μl chloroform and 200 μl Milli-Q water. The mixture was centrifuged (5,000 rpm, 4°C, 5 min). Subsequently, the aqueous layer was filtered using 5-kDa-cutoff filters (Millipore, Bedford, MA) and centrifuged (10,000 rpm, 4°C, 6 h). The filtrate was dried using a vacuum evaporator (4,000 rpm, 4°C, 4 h) and reconstituted in 50 μl Milli-Q water containing spiked internal standards (25 mM [each] 3-aminopyrrolidine and trimesic acid) before analysis.

CE-TOF/MS was performed using an Agilent CE capillary electrophoresis system coupled with an Agilent 6210 time of flight mass spectrometer (Agilent Technologies, Palo Alto, CA) by Human Metabolome Technologies, Inc. (HMT; Tsuruoka, Japan), as described previously (52).

### Bacterial metabolite contamination testing.

Since V. parahaemolyticus can adhere to the host cell surface during the coculture mammalian period, bacterially derived metabolites can thus be coextracted with the host’s metabolites. We determined that after two washing cycles performed with 5 ml of 5% mannitol solution, the abundance of V. parahaemolyticus adhered in Caco-2 cells was approximately 1 × 10^5^ bacterial cells per sample. Still, we were unable to detect significant bacterially derived metabolites in 1 × 10^7^ bacterial cells/ml in our CE-TOF/MS analysis. The results suggest that the level of bacterially derived metabolite contamination was negligible compared to the level of host-derived metabolites.

### Data processing, normalization, and statistical analysis.

Data processing was conducted using Mass Hunter software (Agilent Technologies, Palo Alto, CA). Metabolites are identified by matching their normalized migration times and accurate *m/z* values for each peak to those of the mixture of standard compounds (Human Metabolome Technologies, Inc. [HMT], Tsuruoka, Japan). The peak area values of the metabolites were normalized by median centering. Missing values were replaced by the lowest value detected for that specific metabolite. Statistical analyses of fold changes by two-way analysis of variance (ANOVA) and Dunnett’s multiple-comparison tests were used to identify metabolites with a significant alteration (*P ≤ *0.05) for each group of samples infected by a different V. parahaemolyticus strain. Statistical analysis was performed using GraphPad Prism 8 (GraphPad Software, USA). Multivariate statistical analysis, hierarchical clustering by heat map, and PCA of metabolite’s data set were computed using the Web-based MetaboAnalyst suite (26). Metabolite Set Enrichment Analysis (MSEA) was performed using MetaboAnalyst 4.0. Data representing the metabolites detected in each of the sample groups were inputted into MSEA with annotations based on standard chemical names. Metabolites were confirmed manually using the Human Metabolome Database (HMDB), and a human reference pathway library was used for the pathway enrichment analysis (KEGG Database).

### Cytotoxicity assay.

The cytotoxicity assay used quantitative measurement of lactate dehydrogenase (LDH) release in the culture medium. In brief, the supernatants of infected cells or treatments were collected at the corresponding postinfection points, and lactate dehydrogenase (LDH) release was measured with a nonradioactive cytotoxicity assay (Promega, Madison, WI) according to the manufacturer’s manual. Fifty microliters of cell culture supernatant was transferred into a transparent 96-well plate, 50 μl of assay buffer was added, and the reaction mixture was then incubated at room temperature in the dark for 30 min. The reaction was stopped by the addition of 50 μl of 1 M stop buffer. LDH release was quantified by measuring absorbance at 490 nm.

### Glutathione measurement.

Total glutathione levels were determined using a glutathione quantification kit (Dojindo Molecular Technologies, Inc.) according to the manufacturer’s instructions. In brief, in six-well plates, Caco-2 cells infected with V. parahaemolyticus strains were washed twice with 2 ml of PBS. The cells were then lysed by the addition of 300 μl of 10 mM HCl and then frozen and thawed twice, and the samples were then centrifuged at 12,000 rpm for 10 min. The supernatant was used for a GSH assay, and the absorbance at 405 nm was measured.

### Reactive oxygen species measurement.

Cells (10^4^/well) were seeded in 96-well plates for 4 days. Cells were infected with different strains of V. parahaemolyticus for the respective periods with an MOI of 50:1. After removal of the culture medium, the level of hydroperoxide was measured by incubation with 5 μM BES-H_2_O_2_-AM and 2.5 μM Bes-SO-AM (Wako Chemicals, Osaka, Japan) at 37°C for 1 h. After two washes with PBS, the BES-H_2_O_2_-AM and Bes-SO-AM fluorescent signals were detected at excitation/emission wavelengths of 485/530 and 505/544 nm, respectively.

### PFK activity measurement.

Phosphofructokinase (PFK) activity was measured using a phosphofructokinase activity colorimetric assay kit (BioVision, Inc., USA) according to the manufacturer’s instructions.
