# Supplementary figures and images for: Type III Secretion Effector VopQ of Vibrio parahaemolyticus Modulates Central Carbon Metabolism in Epithelial Cells
Source: mSphere. 2020 Mar 18;5(2):e00960-19. doi: 10.1128/mSphere.00960-19 (PMC7082145; doi:10.1128/mSphere.00960-19)

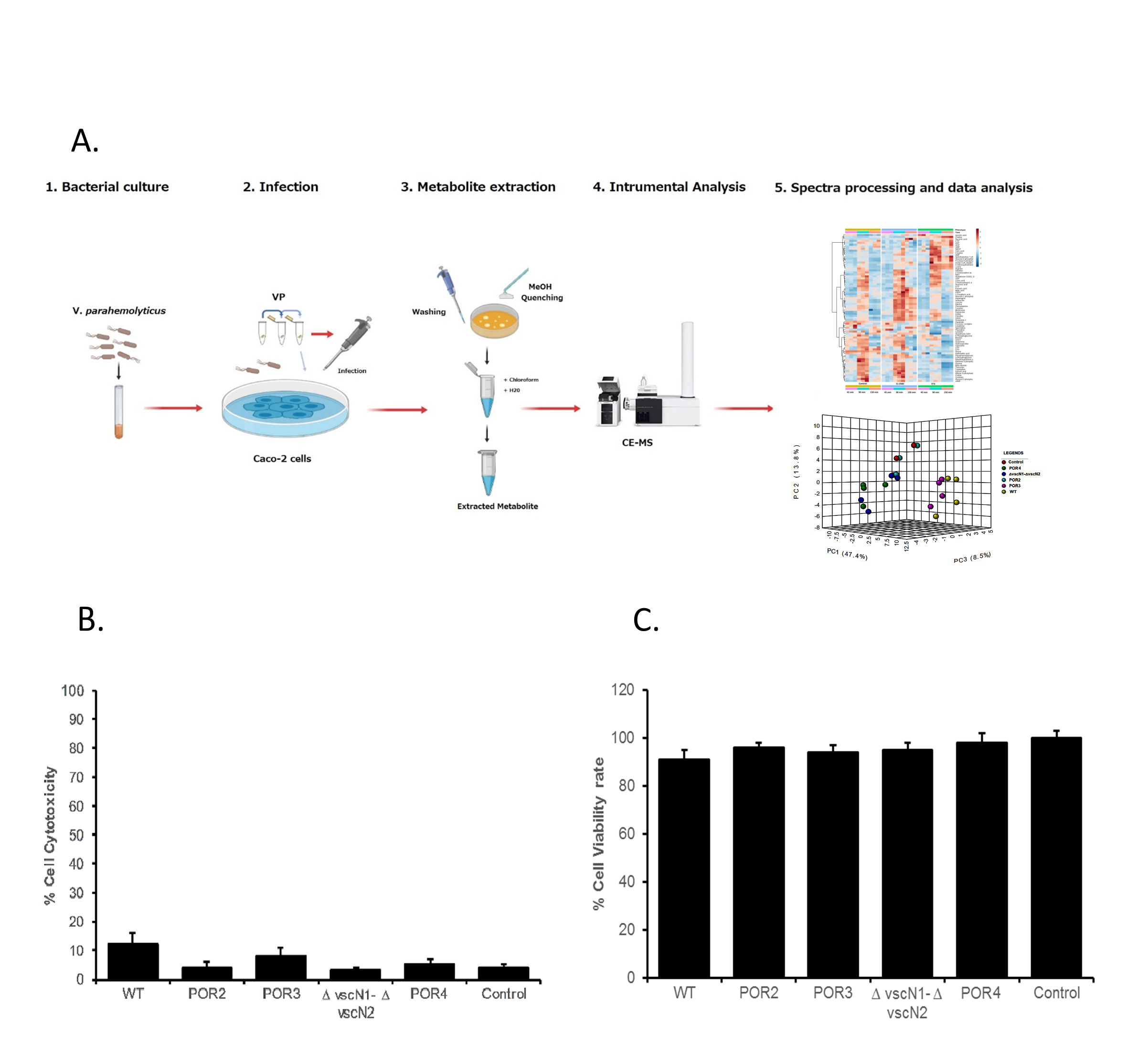

Supplement: FIG S1 [file mSphere.00960-19-sf001.tif]

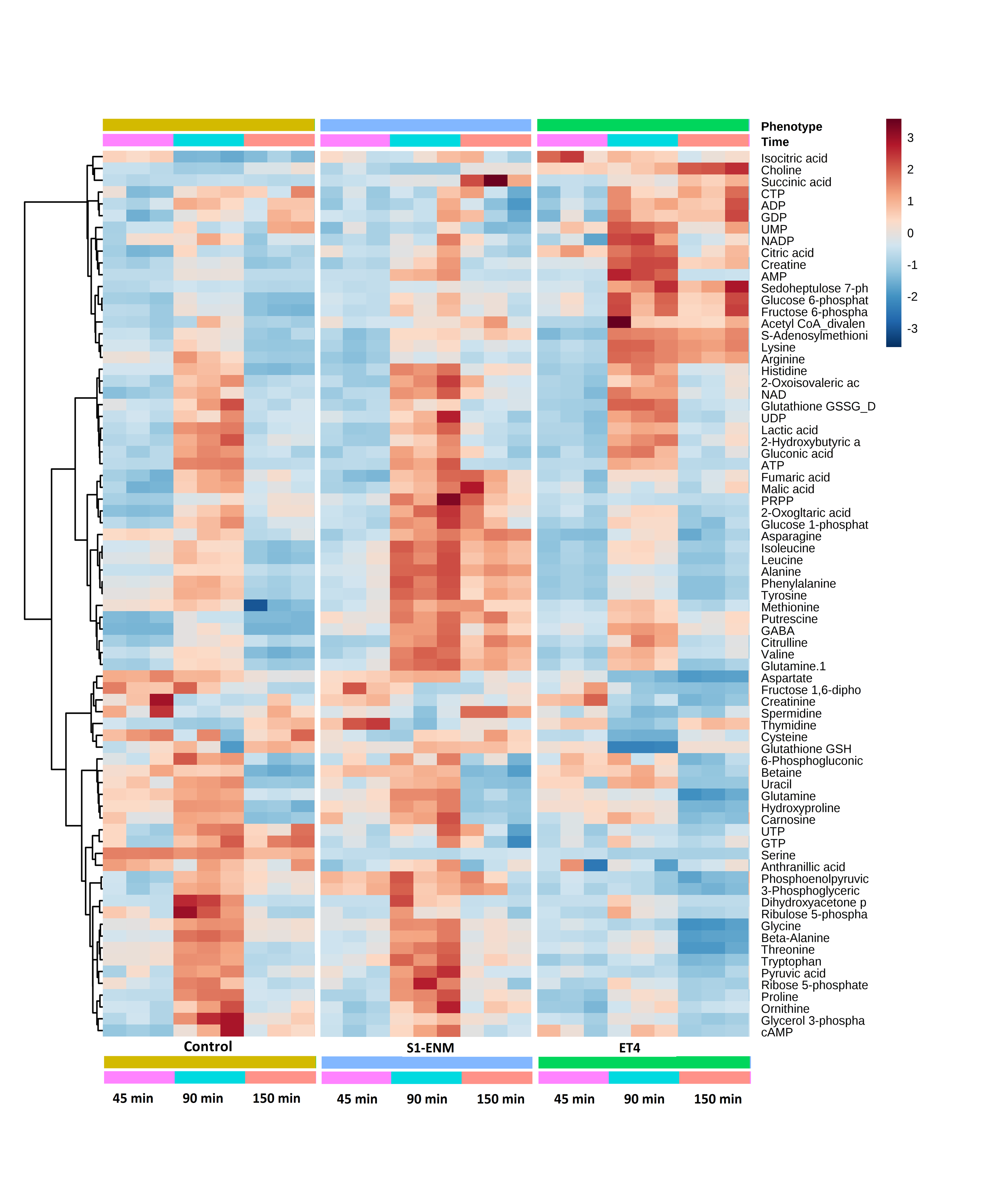

Supplement: FIG S2 [file mSphere.00960-19-sf002.tif]

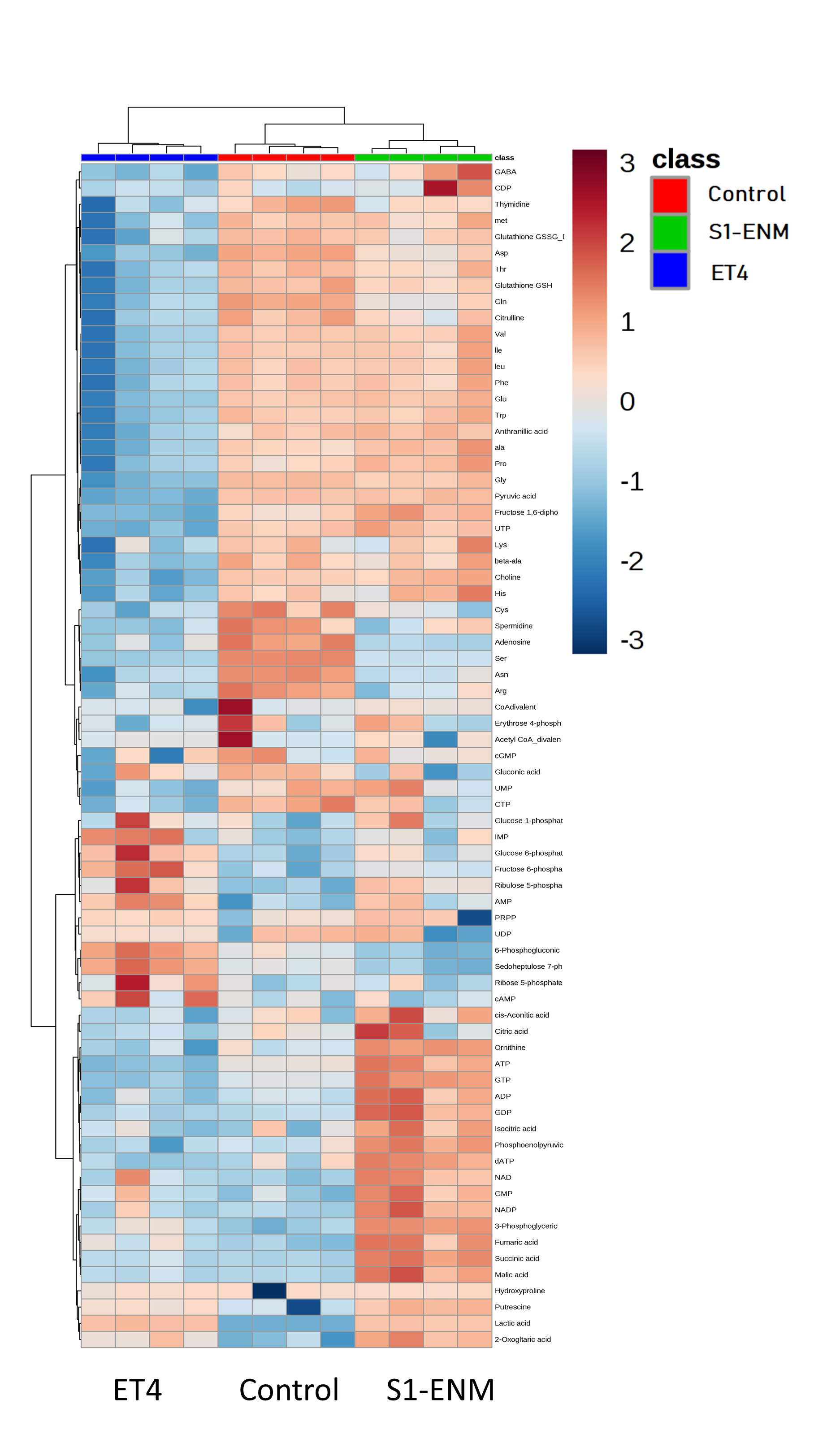

Supplement: FIG S3 [file mSphere.00960-19-sf003.tif]

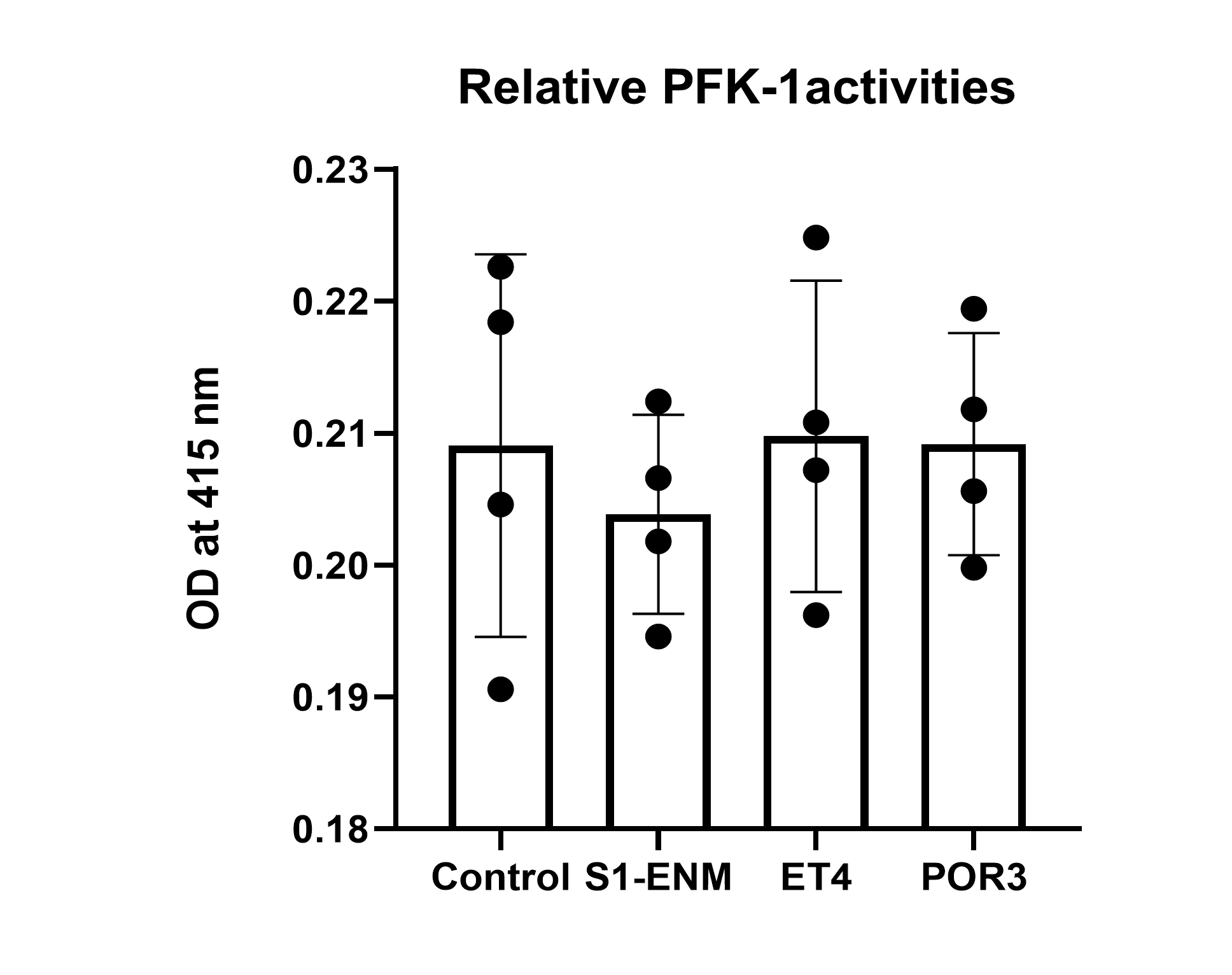

Supplement: FIG S4 [file mSphere.00960-19-sf004.tif]

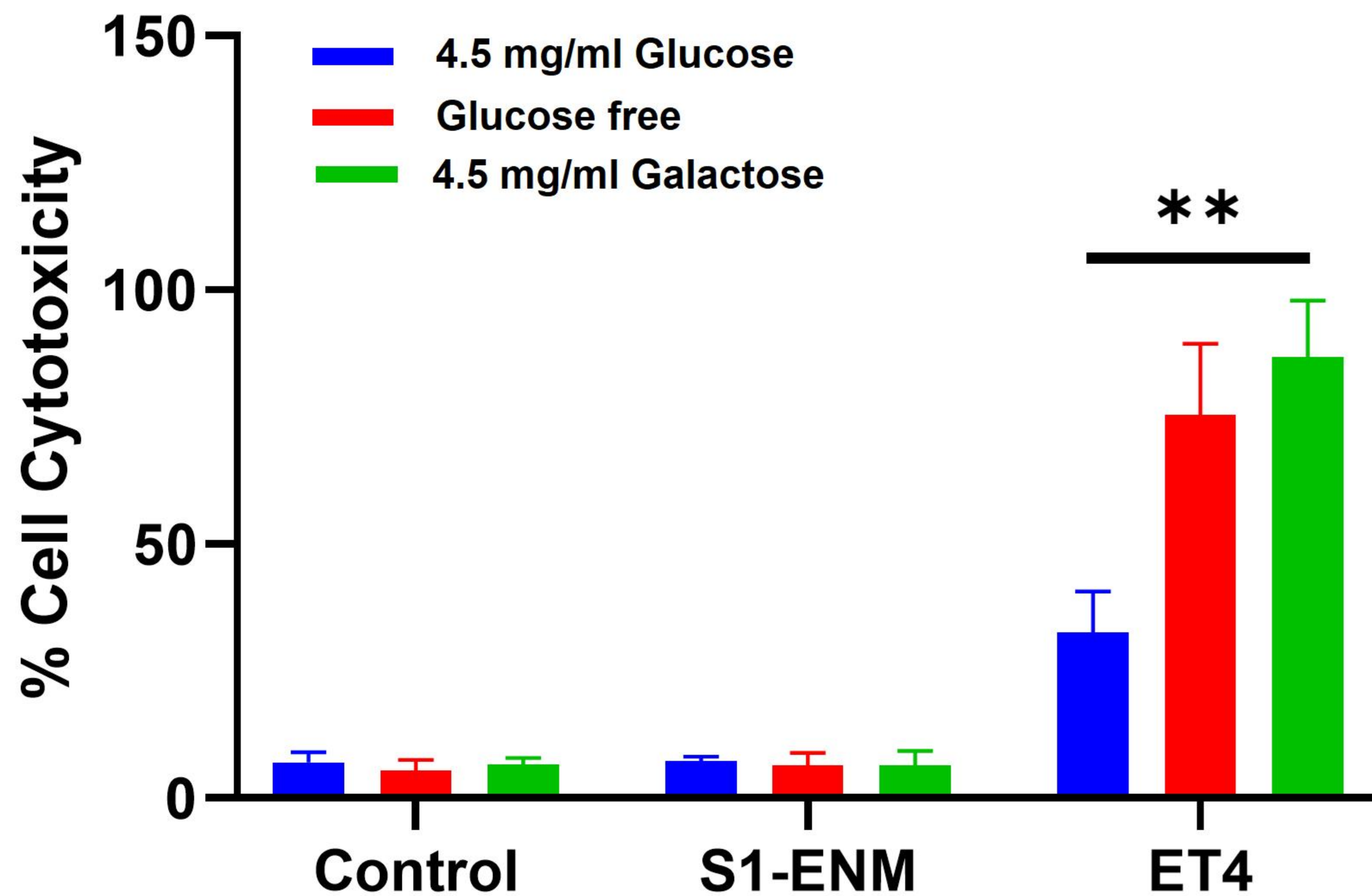

Supplement: FIG S5 [file mSphere.00960-19-sf005.pdf]

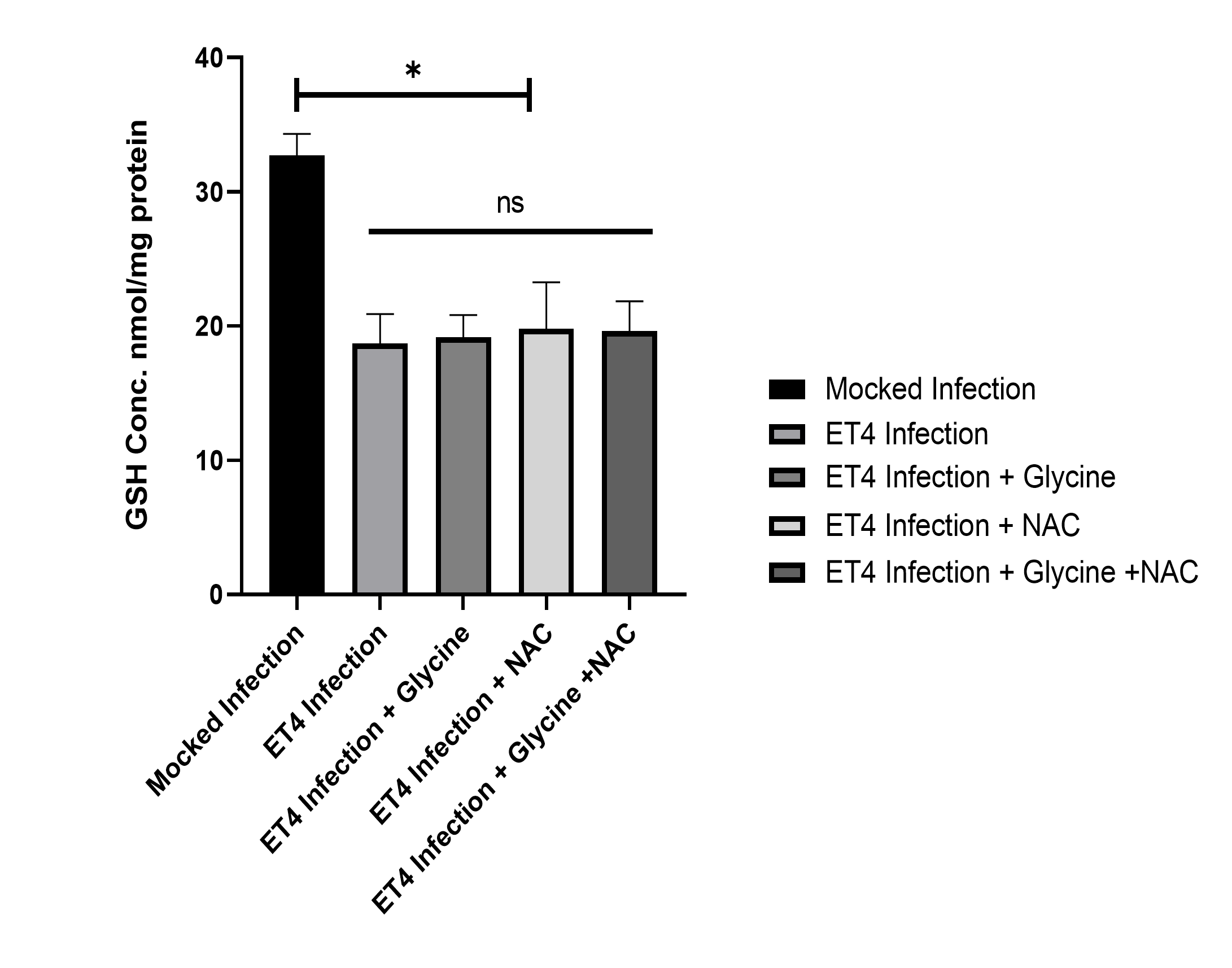

Supplement: FIG S6 [file mSphere.00960-19-sf006.tif]

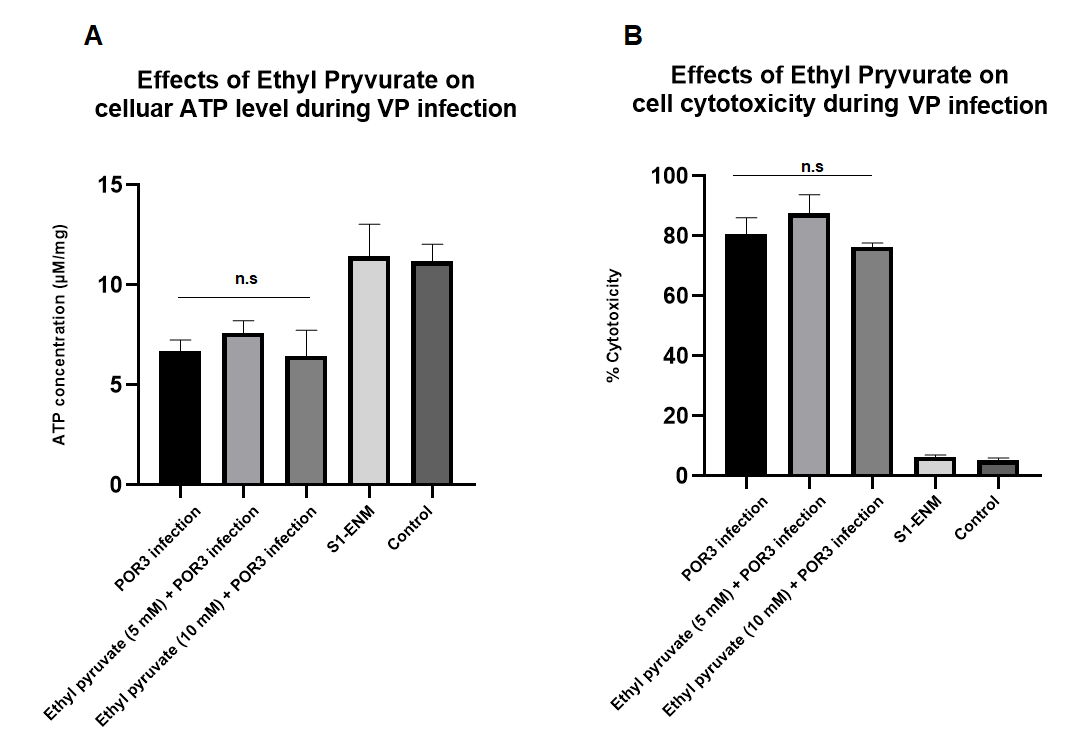

Supplement: FIG S7 [file mSphere.00960-19-sf007.tif]
