# Supplementary material for: Type III Secretion Effector VopQ of Vibrio parahaemolyticus Modulates Central Carbon Metabolism in Epithelial Cells
Source: mSphere. 2020 Mar 18;5(2):e00960-19. doi: 10.1128/mSphere.00960-19 (PMC7082145; doi:10.1128/mSphere.00960-19)
Supplement: TABLE S1 [file mSphere.00960-19-st001.pdf]

No.	Primer target	Primer sequence (5'-3')	Source
**For genetic deletion**			
1.	VP1680-1-F	GGATCCATGATCGATTCGTGCTGATAC	This study
	VP1680-2-R	AATCCAGCCTTCGGCCGTTGTATTCACCAT	
	VP1680-3-F	ATGGTGAATACAACGGCCGAAGGCTGGATT	
	VP1680-4-R	CTGCAGGCAATTTGTGAAAGACAACG	
2	VP1683-1-F	ATCCGGGGATCCGCATCAAATGTGGTCGCTCT	This study
	VP1683-2-R	ACAAGCCTCGATTTATGACGTATTGATATTAACCATTGT	
	VP1683-3-F	TAAATCGAGGCTTGTACGTTG	
	VP1683-4-R	ATCCGGCTGCAGCATCTTCTGTGGCGGTGAAG	
3	VP1686-1-F	GGATCCATGAAAGGGTCGCTCACTCA	This study
	VP1686-2-R	GTCCGCAACTGGATTACTAGCAGCATTGAT	
	VP1686-3-F	ATCAATGCTGCTACTAATCCAGTTGCGGAC	
	VP1686-4-R	CTGCAGAGCGCATAACAATGAGGGAC	
4	VPA0450-1-F	ATCCGGGGATCCATGACCATGATGAAGCGCTT	This study
	VPA0450-2-R	GGCTCTGTGGCCTAAGACTATTAATTTGAATTGTCGACAT	
	VPA0450-3-F	TTAGGCCACAGAGCCGTAT	
	VPA0450-4-R	ATCCGGCTGCAGCGTTAAGGCACCAGAAGCT	
**For complementation**			
1.	VP1680-F-C	GGATCCATGGTGAATACAACGCAAAA	1
	VP1680-R-C	GAATTCTTAAATCCAGCCTTCGGCT	

1. Nakano M, Takahashi A, Su Z, Harada N, Mawatari K, Nakaya Y. 2008. Hfq regulates the expression of the thermostable direct hemolysin gene in *Vibrio parahaemolyticus*. BMC Microbiol 8:155. https://doi.org/ 10.1186/1471-2180-8-155.
